# Downregulated expression of hepatoma-derived growth factor inhibits migration and invasion of prostate cancer cells by suppressing epithelial-mesenchymal transition and MMP2, MMP9

**DOI:** 10.1371/journal.pone.0190725

**Published:** 2018-01-04

**Authors:** Feilong Yang, Nengwang Yu, Hui Wang, Cong Zhang, Zhao Zhang, Yanxiang Li, Dawei Li, Lei Yan, Hainan Liu, Zhonghua Xu

**Affiliations:** 1 Department of Urology, Qilu Hospital of Shandong University, Jinan, P.R. China; 2 Key Laboratory of Cardiovascular Remodeling and Function Research, Shandong University, Jinan, P.R. China; 3 Department of Urology, Dezhou People Hospital, Dezhou, Shandong Province, P.R. China; University of South Alabama Mitchell Cancer Institute, UNITED STATES

## Abstract

Hepatoma-derived growth factor (HDGF) is commonly over-expressed and plays critical roles in the development and progression in a variety of cancers. It has previously been shown that HDGF is overregulated in prostate cancer cells compared to normal prostate cells, which is correlated with cellular migration and invasion of prostate cancer. Here, the molecular mechanisms of HDGF in prostate cancer is investigated. It is shown that HDGF knockdown reduces prostate cancer cellular migration and invasion in both androgen-sensitive LNCaP cells and androgen-insensitive DU145 and PC3 cells. Furthermore, Western blot analysis reveals that HDGF knockdown inhibits epithelial-mesenchymal transition (EMT) of prostate cancer cells by upregulation of protein E-cadherin and downregulation of proteins N-cadherin, Vimentin, Snail and Slug. In addition, mechanistic studies reveal that proteins MMP2 and MMP9 are down-regulated. In conclusion, our data suggested that HDGF knockdown inhibits cellular migration and invasion in vitro of prostate cancer via modulating epithelial-mesenchymal transition (EMT) signaling pathway, as well as MMP2 and MMP9 signaling pathway. These results supported that HDGF is a relevant protein in the progression of prostate cancer and may serve as a potentially therapeutic target for prostate cancer as well as its downstream targets.

## Introduction

Prostate cancer (PCa) develops in the unique gland of the male reproductive system and becomes the most common malignancy in men, which leads to a detriment to men’s health. In 2015, PCa was ranked the second most frequently diagnosed cancer in males worldwide and the fifth leading cause of cancer deaths in the world [1]. In the United States, PCa alone accounts for almost 1 in 5 new dignoses of the most common cancers expected to occur in men, and accounts for 8% of all cancer deaths in men which is the third leading cause of cancer-related deaths for men [2]. Besides, in China, it was estimated that the incidence of prostate cancer was ranked sixth and the mortality of prostate cancer was ranked seventh in men [3]. Currently, there are several effective treatments including surgery, androgen ablation and radiation therapy for hormone dependent PCa, but the curative effect to hormone independent cases is unsatisfactory [4]. Though widely studied, the precise mechanism of PCa has not yet been fully clarified and further investigation is needed.

Hepatoma-derived growth factor (HDGF), an acidic heparin-binding growth factor, was originally purified from the conditioned medium of a human hepatoma cell line, Huh-7 [5]. HDGF is widely expressed in several embryonic tissues including brain, kidney, liver, and cardiovascular system [6–8], which is closely associated with cellular processes including proliferation, differentiation and migration of these fetal cell types [8]. Previous studies have shown that HDGF is highly expressed in numerous cancer tissues and characterized by a novel prognostic factor, such as oral cancer [9], hepatoma [10–12], lung cancer [13, 14], gallbladder cancer [15], pancreatic cancer [16], endometrial carcinoma [17], and gastric carcinoma [18, 19] esophageal carcinoma [20], colorectal cancer [21], and cholangiocarcinoma [22]. Moreover, increasing investigations revealed that HDGF is significantly correlated with the malignant biological potential of a variety of cancer cells including PCa cells [23]. However, the signaling pathway and molecular mechanisms are largely unknown.

Epithelial-mesenchymal transition (EMT), defined as that tumor cells lose the epithelial morphology and acquire mesenchymal characteristics during carcinogenic progression, participates in increased cell migration and invasion, and induces the initial stage of metastatic progression during the development and progression of malignant tumors [24]. Besides, matrix metalloproteinases (MMPs) including MMP2 and MMP9 also contribute to the invasive and metastatic phenotypes of a variety of cancer cells by degrading the extracellular matrix and other barriers [25]. However, it is unknown whether HDGF regulates cell migration and invasion via EMT process or the expression of MMPs in PCa. Therefore, we investigate the role of HDGF knockdown in the regulation of migration and invasion and EMT, as well as MMP2, MMP9 in PCa cells.

## Materials and methods

### Cell culture

Human PCa cells(DU145, PC3 and LNCaP cells) were obtained from the Shanghai Institutes for Biological Sciences in China. DU145 and LNCaP cells were maintained in RPMI-1640 medium containing 10% fetal bovine serum (FBS) purchased from Hyclone, and PC3 cells were cultured in F12K medium containing 10% FBS. All cells were routinely cultured in 5% CO2 at 37°C.

### Lentivirus-mediated shRNA interference

The recombinant lentivirus short hairpin RNA (shRNA) targeting HDGF sequence (shRNA-HDGF) and control shRNA (shRNA-CTR) were purchased from Bio-Link (Shanghai, China). The target sequence of shRNA-HDGF as described by Jun *et al*. [26] was as follows: 5′–AACCGGCAGAAGGAGTACAAA–3′. The scrambled sequence of shRNA-CTR was as follows: 5′–TTCTCCGAACGTGTCACGT–3′. Cells treated by shRNA-CTR or PBS were used as controls. In vitro transfection were done following the manufacturer’s protocols.

### Quantitative real time PCR

Total RNAs were extracted from the cells using TRIzol (Invitrogen, Carlsbad, CA, USA) according to the manufacturer’s instructions. For the detection of HDGF mRNA levels, reverse transcription and quantitative real time PCR (qRT-PCR) was performed on Prism 7500 (ABI, Foster City, CA, USA) by using standard SYBR green assay protocol. Primers for HDGF is 5′- CTCTTCCCTTACGAGGAATCCA -3′ (forward) and 5′- CCTTGACAGTAGGGTTGTTCTC -3′ (reverse). Primers for β-actin used as normalization control is 5′- CATGTACGTTGCTATCCAGGC -3′ (forward) and 5′- CTCCTTAATGTCACGCACGAT -3′ (reverse). The relative level of HDGF mRNA was calculated and normalized using the 2^-ΔΔCt^ method relative to β-actin.

### Cell scratch assay

All cells were plated in 6-well plates and incubated until 70% - 80% confluent. Vertical scratches were then made using a 200μl plastic filter tip to create a ‘wound’. To eliminate dislodged cells, culture medium was removed and wells were washed with PBS. ‘Wound closure’ was observed at 0, 12, 24 hours and digital images were taken under an inverted microscope. The wound area was measured using Image J software to calculate fold change of area coverage in the wells.

### Cell migration assay

Transwell chamber was used to examine cell migration ability. Cells (1.0×10^5^ cells /well) were placed into the upper chambers (BD Bioscience, San Jose, CA, USA) containing 10% FBS. The lower chambers were filled with RPMI-1640 or F12K with 20% FBS. After incubation for 12 h for DU145 cells, 24 h for PC3 cells, and 48 h for LNCaP cells at 37°C, cells on the lower surface of the membranes were fixed and stained with 0.1% crystal violet, and five random fields of vision were selected to be counted under a light microscope (Olympus). Each experiment was performed in triplicate.

### Cell invasion assay

The invasion assay was similar with the migration, except for that the upper chamber was precoated with matrigel (BD Bioscience, San Jose, CA, USA). In addition, the incubation time for DU145 cells, PC3 cells and LNCaP cells is 24 h, 48 h, and 72 h, respectively.

### Western blotting

Cells were lysed in RIPA buffer (Beyotime, Shanghai, China). Protein concentration was quantified with BCA Protein Quantitative Kit (Beyotime, Shanghai, China). Equal amount of protein (20ug) was separated on 10% SDS-PAGE and transferred to PVDF membranes (Millipore, Billerica, MA, USA), which were blocked with 5% non-fat milk for 1 hour and incubated overnight at 4°C with rabbit anti-human primary antibodies against HDGF, E-cadherin, N-cadherin, Snail, Slug, MMP2, MMP9 and β-actin (all 1:1000 dilution) followed by incubation with HRP-conjugated goat anti-rabbit secondary antibody (1:1000 dilution) for 1 hour and detected by ECL.

### Statistical analysis

Data were expressed as the mean ±SD from at least three independent experiments. The Student’s t-test was used to compare the differences between two groups with SPSS 22.0. *P* < 0.05 was considered statistically significant.

## Results

### Relative HDGF mRNA levels were inhibited by lentivirus-mediated shRNA interference in DU145, PC3 and LNCaP cells

**Fig 1:**
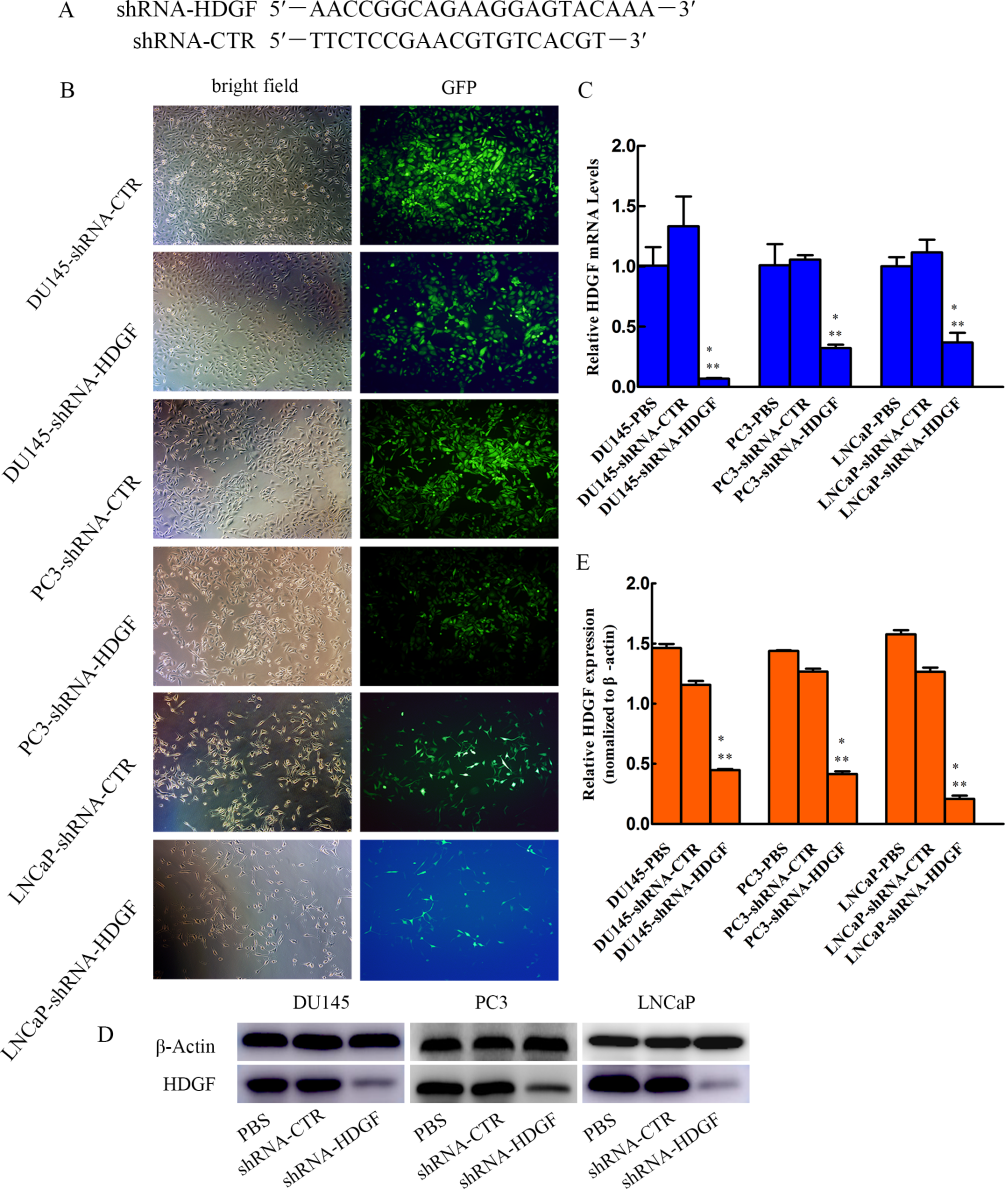
HDGF expression is down-regulated in PCa cells. (A) Sequence of shRNA-HDGF and shRNA-CTR. (B) Lentivirus transfection efficiency. Fluorescence microscopy (magnification, × 200) demonstrated >80% PCa cells were effectively transfected with shRNA-HDGF and shRNA-CTR lentivirus 24 h after transfection at a MOI of 50 for DU145 and PC3 cells respectively, and 36 h after transfection at a MOI of 20 for LNCaP cells. (C) QRT-PCR analysis revealed that HDGF mRNA levels were down-regulated in DU145, PC3 and LNCaP cells. The relative gene expression level of HDGF was normalized to β-actin. (D, E) Expression of HDGF protein in human PCa cells (DU145, PC3 and LNCaP cells) transfected with lentivirus shRNA-HDGF is reduced compared with the corresponding cells transfected with lentivirus shRNA-CTR or only treated with PBS, and the quantification of HDGF gray intensity of DU145, PC3 and LNCaP cells in each group was presented. The data are presented as mean ± standard deviation of three independent experiments. *P < 0.05 vs PBS, **P < 0.05 vs shRNA-CTR. PCa, prostate cancer; MOI, multiplicity of infection; HDGF, hepatoma-derived growth factor; shRNA-HDGF, group transfected by recombinant lentivirus shRNA targeting HDGF sequence; shRNA-CTR, group transfected by recombinant lentivirus shRNA with scrambled sequence; PBS, group treated by PBS.

DU145, PC3 and LNCaP cells were transfected by lentivirus shRNA-HDGF or shRNA-CTR. To investigate the transfection efficiency, primarily we examined the expression of Green Fluorescence Protein (GFP). The transfection efficiency of >80% was determined by detecting the expression of GFP 24 h after transfection at a MOI of 50 for DU145 and PC3 cells, and 36 h after transfection at a MOI of 20 for LNCaP cells (Fig 1B). Then the cells were cultured in their medium with puromycin to select cells transfected stably for later HDGF knockdown experiments. Further, we analyzed the HDGF mRNA levels in the selected cells by quantitative real time PCR analysis (Fig 1C). Results of this analysis showed that the relative levels of HDGF mRNA were suppressed in DU145, PC3 and LNCaP cells treated by lentivirus shRNA-HDGF compared with these cells treated by lentivirus shRNA-CTR or PBS.

### Expression of HDGF was inhibited by lentivirus-mediated shRNA interference in DU145, PC3 and LNCaP cells

Protein is significantly correlated with the function of cells. To investigate whether the expression of HDGF was knocked down successfully, we examined the protein level of HDGF by western blotting analysis. Results of this analysis suggested that the relative protein expression levels of HDGF were significantly downregulated in DU145, PC3 and LNCaP cells treated by lentivirus shRNA-HDGF compared with these cells treated by lentivirus shRNA-CTR or PBS (Fig 1D and 1E).

### HDGF downregulation reduces PCa cell invasion and migration in vitro

**Fig 2:**
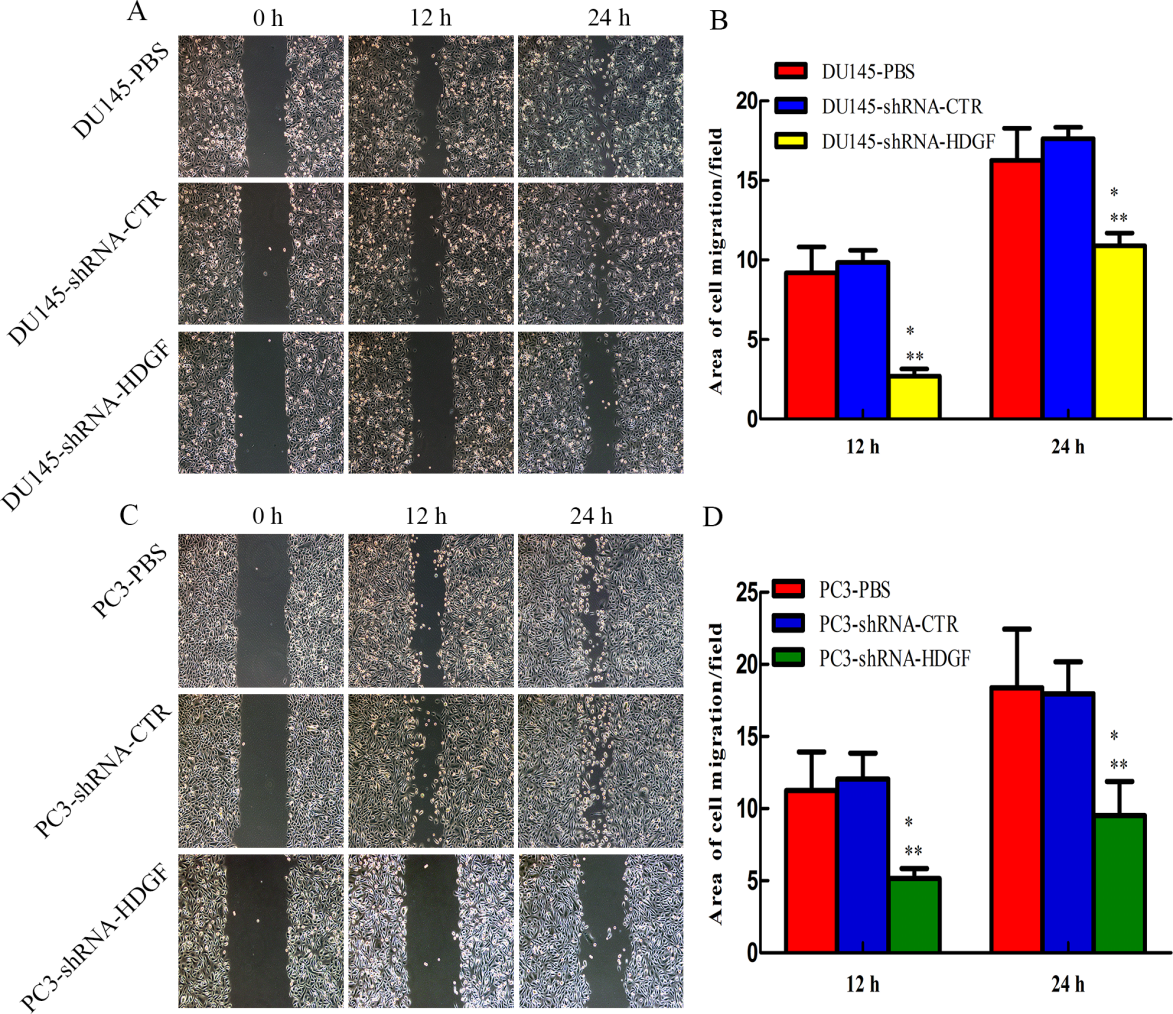
Effect of HDGF knockdown on motility of DU145 and PC3 cells. Scratch assays showed that HDGF knockdown reduced the area of migration of (A, B) DU145 and (C, D) PC3 cells respectively compared with cells transfected by lentivirus shRNA-CTR or treated by PBS. Representative images (magnification, × 200) and quantification of mean area of cell migration of DU145 and PC3 cells in each group are presented. The data are presented as mean ± standard deviation of three independent experiments. *P < 0.05 vs PBS, **P < 0.05 vs shRNA-CTR. PCa, prostate cancer; HDGF, hepatoma-derived growth factor; shRNA-HDGF, group transfected by recombinant lentivirus shRNA targeting HDGF sequence; shRNA-CTR, group transfected by recombinant lentivirus shRNA with scrambled sequence; PBS, group treated by PBS.

**Fig 3:**
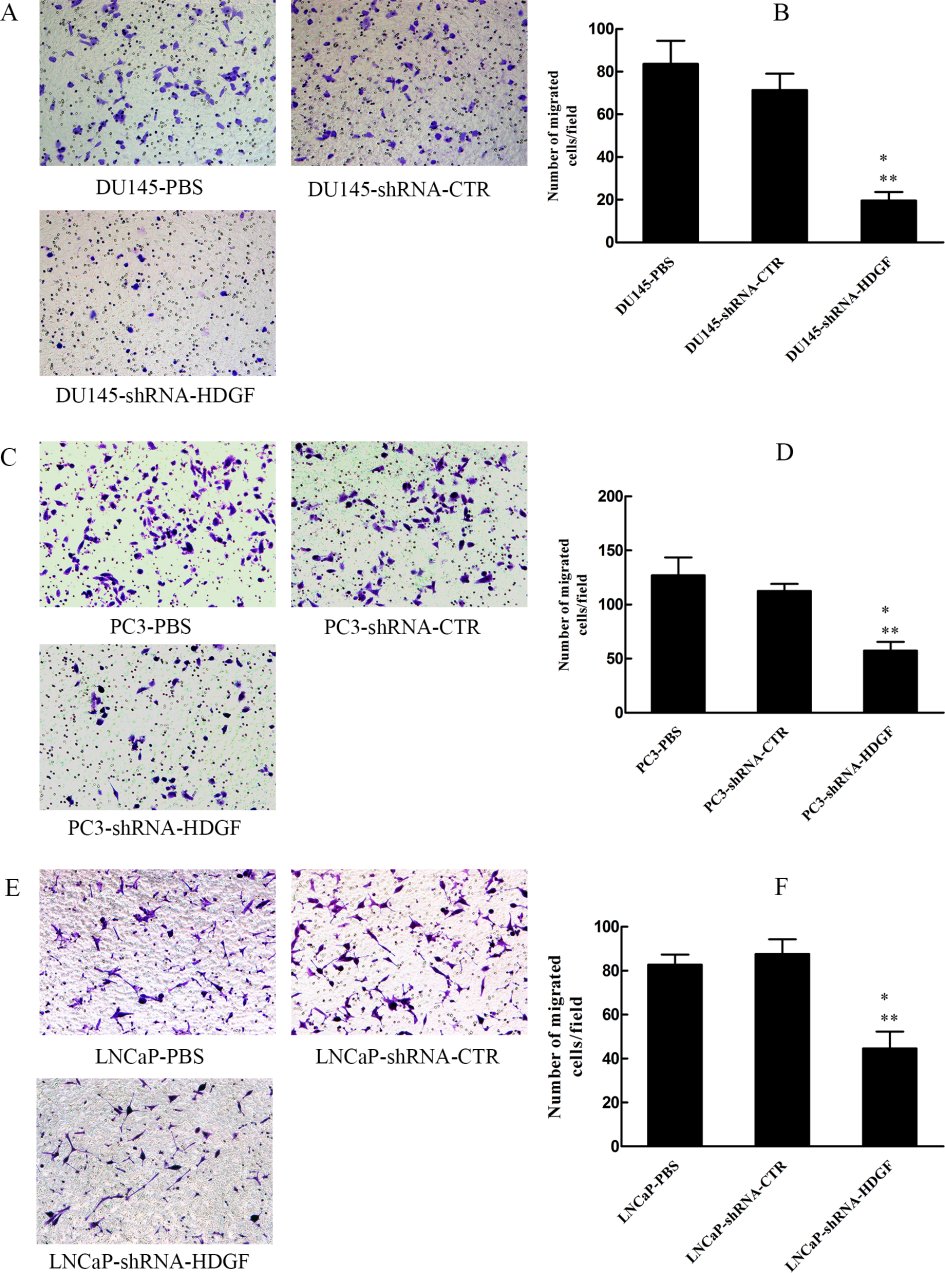
Effect of HDGF knockdown on PCa cell migration. Cell migration capacity of transfected PCa cells by a Transwell assay and a quantification of the number of migrated (A, B) DU145, (C, D) PC3 and (E, F) LNCaP cells in each group. Representative images (magnification, × 200) and a quantification of mean migration number of DU145, PC3 and LNCaP cells in each group are presented. The data are presented as mean ± standard deviation of three independent experiments. *P < 0.05 vs PBS, **P < 0.05 vs shRNA-CTR. PCa, prostate cancer; HDGF, hepatoma-derived growth factor; shRNA-HDGF, group transfected by recombinant lentivirus shRNA targeting HDGF sequence; shRNA-CTR, group transfected by recombinant lentivirus shRNA with scrambled sequence; PBS, group treated by PBS.

**Fig 4:**
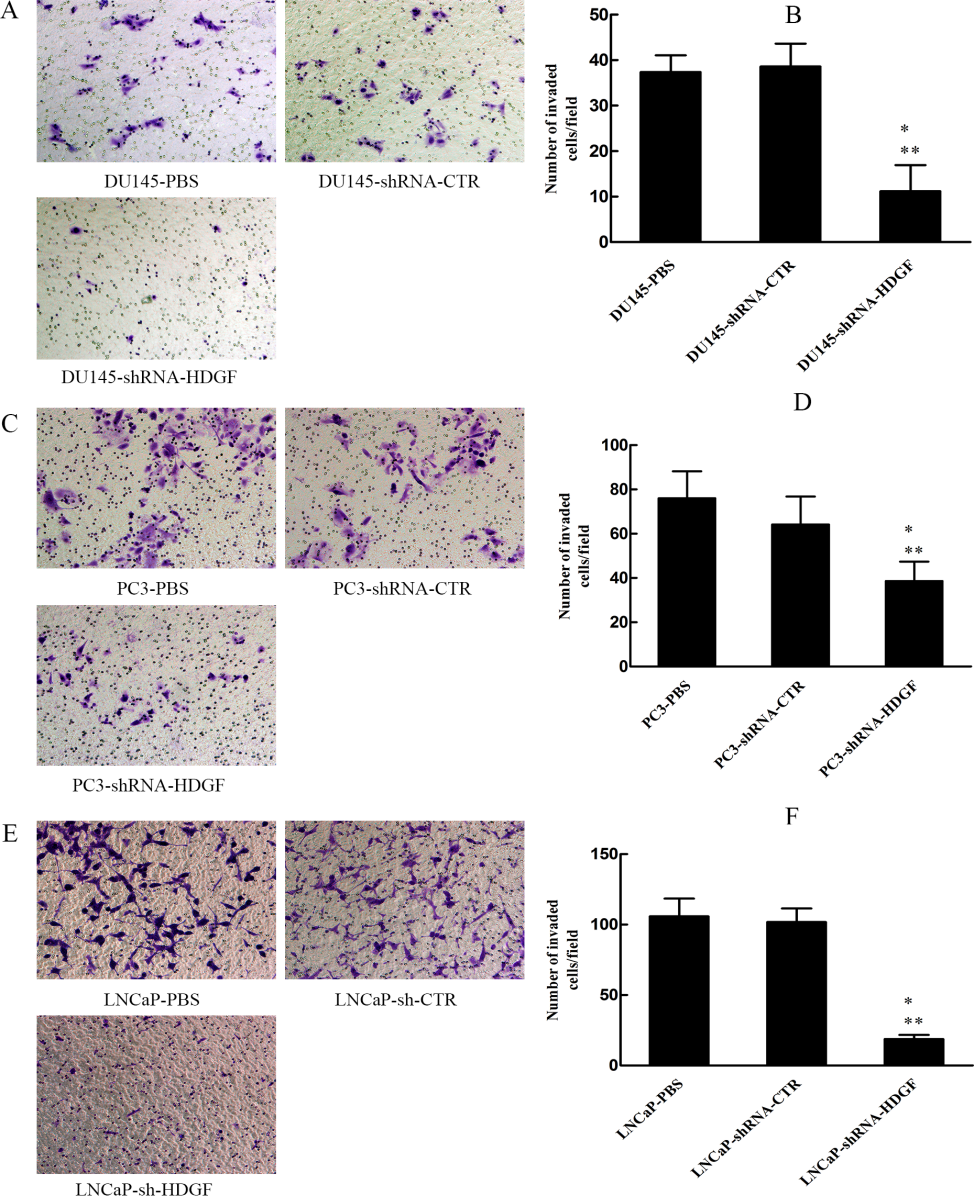
Effect of HDGF knockdown on PCa cell invasion. Cell invasion capacity of transfected PCa cells by a Transwell assay with Matrigel and a quantification of the number of invaded (A, B) DU145, (C, D) PC3 and (E, F) LNCaP cells in each group. Representative images (magnification, × 200) and quantification of mean invasion number of DU145, PC3 and LNCaP cells in each group are presented. The data are presented as mean ± standard deviation of three independent experiments. *P < 0.05 vs PBS, **P < 0.05 vs shRNA-CTR. PCa, prostate cancer; HDGF, hepatoma-derived growth factor; shRNA-HDGF, group transfected by recombinant lentivirus shRNA targeting HDGF sequence; shRNA-CTR, group transfected by recombinant lentivirus shRNA with scrambled sequence; PBS, group treated by PBS.

Since tumorigenesis is involved of multiple oncogenic processes including migration and invasion, we examined whether modulation of HDGF expression affected these processes. The effects of HDGF on the migration of PCa cells were assessed by scratch assay and migration assay, while the effect of HDGF on the invasion of PCa cells was assessed by Matrigel invasion assay. The results of scratch assay demonstrated that compared with lentivirus shRNA-CTR transfected cells, HDGF knockdown significantly reduced the area of migration of DU145 (Fig 2A and 2B) and PC3 (Fig 2C and 2D) cells respectively. Besides, The results of migration assay demonstrated that HDGF knockdown significantly reduced the number of migrated DU145 (Fig 3A and 3B), PC3 (Fig 3C and 3D) and LNCaP (Fig 3E and 3F) cells compared with lentivirus shRNA-CTR transfected cells or PBS treated cells. Meanwhile, The results of the Matrigel invasion assay indicated that inhibition of HDGF reduced the number of invaded DU145 (Fig 4A and 4B), PC3 (Fig 4C and 4D) and LNCaP (Fig 4E and 4F) cells compared with lentivirus shRNA-CTR transfected cells or PBS treated cells. These findings provided evidence that inhibited HDGF expression levels were involved in suppressing the migratory and invasive ability of PCa cells.

### Epithelial-mesenchymal transition (EMT)-associated proteins in PCa cells

**Fig 5:**
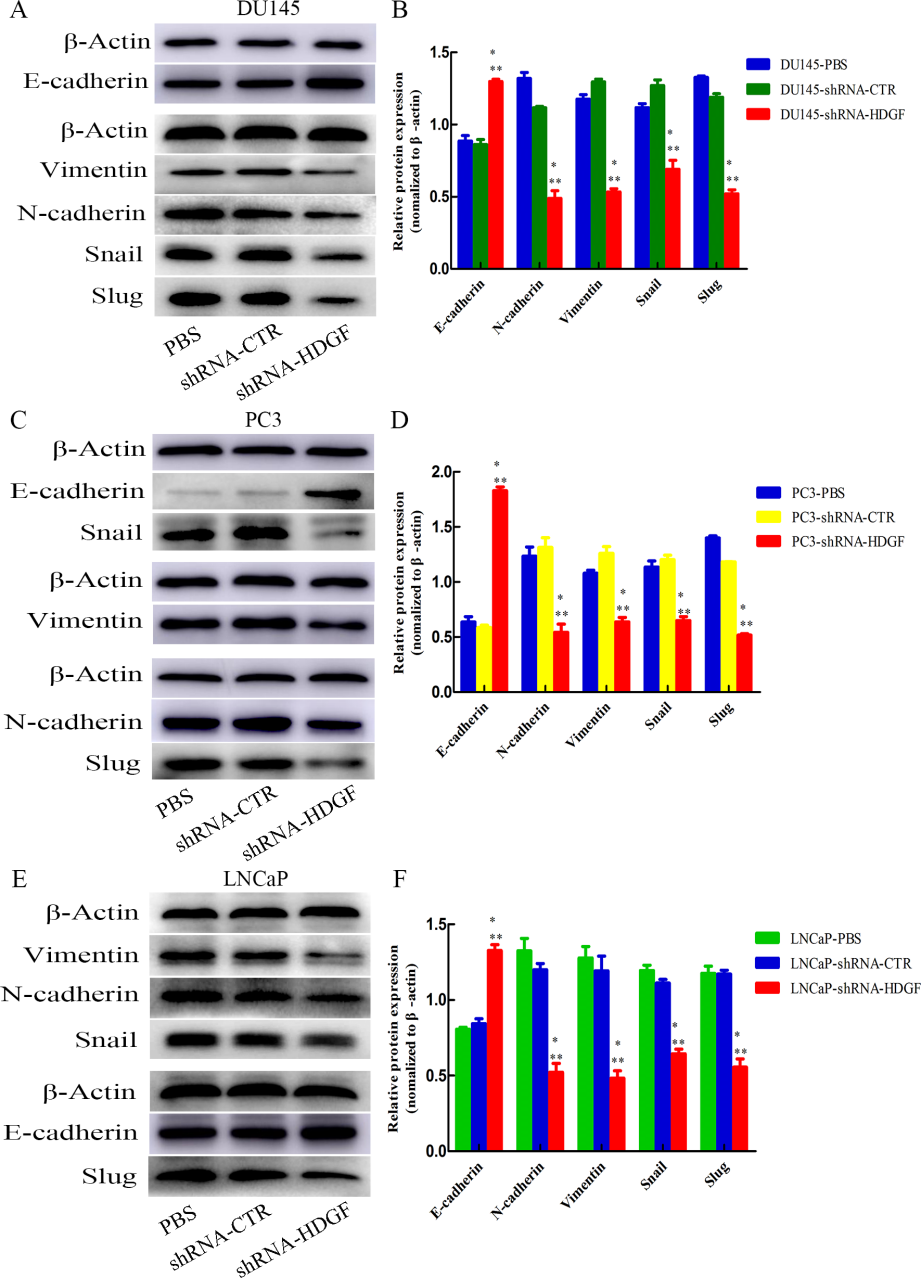
Expression levels of EMT-associated proteins in PCa cells following knockdown of HDGF. The relative protein expression levels of Vimentin, N-cadherin, Snail and Slug were reduced, and E-cadherin was increased in shRNA-HDGF transfected (A) DU145, (C) PC3 and (E) LNCaP cells compared with their corresponding control cells transfected with shRNA-CTR or treated with PBS. The quantification of EMT-associated protein gray intensity of (B) DU145, (D) PC3 and (F) LNCaP cells in each group are presented. The data are presented as mean ± standard deviation of three independent experiments. The relative protein expression level was normalized to β-actin. *P < 0.05 vs PBS, **P < 0.05 vs shRNA-CTR. EMT, epithelial mesenchymal transition; PCa, prostate cancer; HDGF, hepatoma-derived growth factor; shRNA-HDGF, group transfected by recombinant lentivirus shRNA targeting HDGF sequence; shRNA-CTR, group transfected by recombinant lentivirus shRNA with scrambled sequence; PBS, group treated by PBS.

To further investigate the mechanism by which HDGF regulates cell invasion and migration, the protein expression levels of EMT-associated genes in PCa cells was analyzed. It was demonstrated that HDGF knockdown decreased the expression of the mesenchymal markers Vimentin and N-cadherin, and the EMT central regulators Snail and Slug compared with shRNA-CTR transfected cells or PBS-treated cells, while the expression of epithelial marker E-cadherin was upregulated in PCa cell lines DU145(Fig 5A and 5B), PC3 (Fig 5C and 5D) and LNCaP (Fig 5E and 5F) following HDGF knockdown. Thus, the results of the current study demonstrated that knockdown of HDGF altered the expression levels of EMT-associated proteins, therefore, suppressing the invasive and migratory phenotype of PCa cells.

### Effects of HDGF knockdown on the expression of MMP2 and MMP9

**Fig 6:**
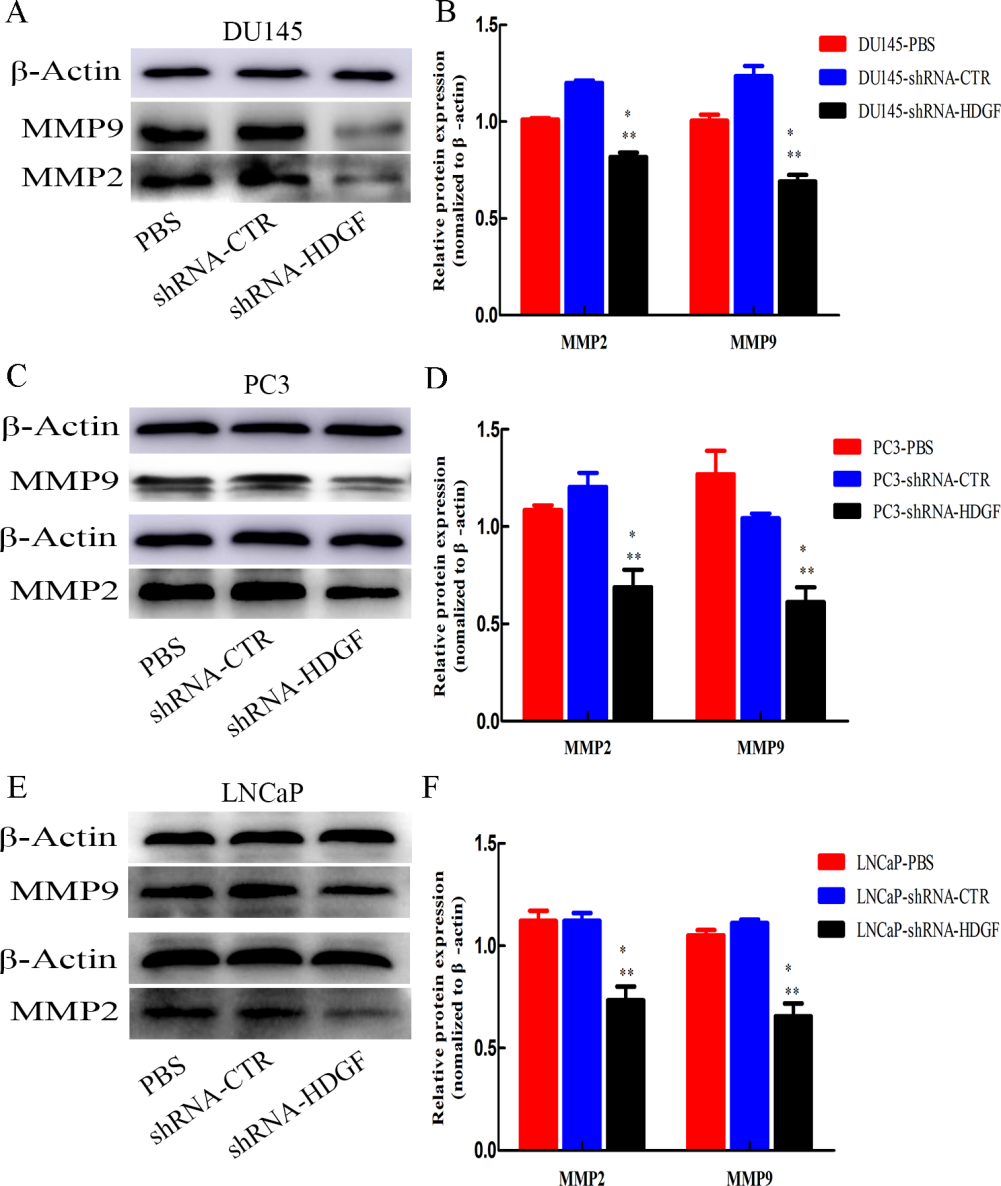
Expression levels of MMPs in PCa cells following knockdown of HDGF. The relative protein levels of MMP2 and MMP2 were decreased in sh-HDGF transfected (A) DU145, (C) PC3 and (E) LNCaP cells compared with their corresponding control cells transfected with shRNA-CTR or treated with PBS. The gray intensity quantification of MMP2 and MMP9 of (B) DU145, (D) PC3 and (F) LNCaP cells in each group are presented. The data are presented as mean ± standard deviation of three independent experiments. *P < 0.05 vs PBS, **P < 0.05 vs shRNA-CTR. PCa, prostate cancer; MMP, matrix metalloproteinase; HDGF, hepatoma-derived growth factor; shRNA-HDGF, group transfected by recombinant lentivirus shRNA targeting HDGF sequence; shRNA-CTR, group transfected by recombinant lentivirus shRNA with scrambled sequence; PBS, group treated by PBS.

As the invasive ability of tumor cells is often correlated with the production of secretory proteases, the effect of HDGF knockdown on the expression of tumor invasion-associated secretory proteases MMPs including MMP2 and MMP9 were determined. Western blotting analysis indicated that downregulation of HDGF reduced the protein expression levels of MMP2 and MMP9 in PCa cells DU145 (Fig 6A and 6B), PC3 (Fig 6C and 6D) and LNCaP (Fig 6E and 6F).

## Discussion

HDGF, a member of the hepatoma-derived growth factor family, is located on chromosome 1, region q21-q23 and encodes 240 amino acids, which contains two putative bipartite nuclear localization sequences (NLSs) for nuclear localization and an important PWWP domain in the N-terminal region for binding DNA [27]. HDGF is a multifunctional protein which involves in the many cellular events, such as RNA processing, DNA damage repair, ribosome biogenesis, and transcriptional regulation [12]. Previous studies reveals that HDGF promotes the proliferation of several types of normal cells such as fibroblast, hepatocyte, endothelial cells, vascular smooth muscle cells [5, 6], and takes part in many cellular physiological processes, such as lung remodeling, sensitization to irradiation, neurotrophic effects, vascular injury, and cardiovascular differentiation [28].

As a mitogen, HDGF is highly expressed in the early developmental stage of several organs and decreases rapidly after birth [6–8], however, several findings recently suggested that HDGF is highly expressed in a variety of cancers [9, 10, 13, 14, 16, 18–22], which seems to suggest that HDGF plays an important role in the tumorigenesis and cancer progression. It has shown that HDGF stimulates the growth of several types of cancer cells such as HuH-7 hepatoma cells, gastric cancer cells [29] and melanoma. Moreover, HDGF involves in the aggressive biological properties, including invasiveness [26], angiogenesis [28] and metastasis [18] of cancer cells. In PCa, HDGF is overexpressed in DU145, PC3 and LNCaP cells compared with the normal prostate cell RWPE-1 [30] and down-regulation of HDGF expression inhibits the proliferation of PCa DU145, PC3 and LNCaP cells [23, 30], and the migration and invasion of DU145 cells [23]. Moreover, up-regulation of HDGF expression in normal RWPE-1 prostate cell facilitates the migration and invasion of RWPE-1 cells [30]. However, the underlying molecular mechanism associated with HDGF-mediated cell migration and invasion in PCa remains elusive.

Since tumorigenesis is related with multiple oncogenic processes including proliferation, anchorage-independent growth, migration and invasion, and our recent study has demonstrated that down-regulation of HDGF expression inhibits proliferation of DU145, PC3 and LNCaP cells, and migration and invasion of DU145 cells [23, 30], we investigated the effects of HDGF knockdown on the migration and invasion of DU145, PC3 and LNCaP cells. As a result, the scratch assay demonstrated HDGF knockdown significantly reduced the area of migration of DU145 and PC3 cells, and the migration assay demonstrated that HDGF knockdown significantly reduced the number of migrated DU145, PC3 and LNCaP cells, and the Matrigel invasion assay indicated that inhibition of HDGF reduced the number of invaded DU145, PC3 and LNCaP cells. These findings revealed that inhibited HDGF expression levels were involved in suppressing the migratory and invasive ability of PCa cells.

However, it is unknown how HDGF affect cell migration and invasion in PCa. Our recent study demonstrated that cancer cell migration and invasion is partly dependent on EMT process and matrix MMPs such as MMP2 and MMP9 [24, 25]. Moreover, HDGF, a secreted growth factor, is correlated with EMT process in breast cancer cells [24]. Usually, EMT process is regulated through the transcription repressors Snail, Slug, Twist and ZEB1, and initiated by the down-regulation of epithelial genes such as E-cadherin, and the up-regulation of mesenchymal genes such as Vimentin and N-cadherin. Previous study shown that EMT is involved in increased cancer cell invasion and facilitates the initial stage of metastatic progression, because they lose the epithelial morphology characterized by down-regulation of E-cadherin expression and acquire the mesenchymal characteristics characterized by up-regulation of Vimentin and N-cadherin expression during carcinogenic progression [24]. In addition, extracellular matrix degradation is also known to be a major step during cancer progression [31]. Previous studies have shown that MMPs including MMP2 and MMP9, which are families of zinc and calcium dependent endopeptidases, contribute to extracellular matrix remodeling [31]. It has revealed that MMP2 and MMP9 are correlated with the invasive and metastatic phenotypes of prostate cancer cells [31]. However, whether HDGF participates in the progression of PCa mediated by EMT and MMPs is largely unknown.Previous studies have shown that the nuclear targeting of HDGF is essential for its translocation to the nucleus and functioning as a direct DNA binding protein to regulate gene transcriptions [32]. In the present study, after examining the inhibiting effect of HDGF knockdown on migration and invasion of Pca cells, we then investigated the molecular events associated with EMT and the expression level of MMPs, mainly including MMP2 and MMP9 after stable HDGF knockdown in PCa cells DU145, PC3 and LNCaP. Consequently, Western blot showed that the expression of transcription repressors Snail and Slug, as well as the mesenchymal markers Vimentin and N-cadherin, were all downregulated (P < 0.05), and the epithelial cell marker E-cadherin were upregulated (P < 0.05). This EMT process was partly verified in breast cancer cells with counterevidence, in which HDGF overexpression stimulates the cell invasion and metastasis by decreasing E-cadherin expression and increasing Vimentin expression [24]. In addition, the expression level of MMP2 and MMP9 in PCa cells were both downregulated (P < 0.05) after stable HDGF knockdown. All these evidence suggested that HDGF downregulated stably by targeting shRNA could inhibit the migration, invasion and EMT process, as well as the expression level of MMP9 and MMP2 in PCa cells. In EMT process, HDGF knockdown may suppressed the expression of transcription repressors Snail and Slug, and further stimulates the expression of E-cadherin and inhibits the expression of Vimentin and N-cadherin, which may result in the inhibition of PCa cell migration and invasion. Moreover, the inhibition of PCa cell migration and invasion may be also partly caused by the down-expression of MMP9 and MMP2 after HDGF knockdown stably.

In conclusion, the downregulation of HDGF significantly is associated with the inhibition of the migration and invasion in prostate cancer cells, at least in part, by suppressing EMT process and the expression of MMP9 and MMP2, which play an important role in the tumorigenesis of PCa. Therefore, HDGF and its downstream molecular events, such as EMT process and the expression of MMP2 and MMP9 may serve as a novel therapeutic target for PCa.

## Supporting information

S1 File S data set. The main data of this study is included in this “pzf” document.
(PZF)
